# 1-(1-Hydr­oxy-9*H*-carbazol-2-yl)-3-methyl­but-2-en-1-one

**DOI:** 10.1107/S1600536810000322

**Published:** 2010-01-09

**Authors:** Matthias Zeller, Makuteswaran Sridharan, Karnam J. Rajendra Prasad, Aimable Ngendahimana

**Affiliations:** a Department of Chemistry, Youngstown State University, One University Plaza, Youngstown, OH 44555, USA; b Department of Chemistry, Bharathiar University, Coimbatore 641 046, Tamil Nadu, India

## Abstract

The title compound, C_17_H_15_NO_2_, was prepared as one of two products of the AlCl_3_/POCl_3_-catalysed reaction of 9-carbazol-1-ol with 3,3-dimethyacrylic acid. It crystallizes with two crystallographically independent mol­ecules, *A* and *B*, which are virtually superimposable but not related by any translational or other pseudosymmetry. Both independent mol­ecules are almost planar [r.m.s. deviations from planarity = 0.053 (1) and 0.079 (1) Å in *A* and *B*, respectively] and contain an intramolecular O—H⋯O hydrogen bond. Each type of mol­ecules is connected *via* pairs of N—H⋯O hydrogen bonds, forming centrosymmetric *A*_2_ and *B*_2_ dimers which are, in turn, arranged in offset π-stacks extending along the *a*-axis direction. The offset of the dimers and the tilt angle of the mol­ecules allows the formation of alternating C—H⋯π inter­actions between *A* and *B* mol­ecules of parallel stacks.

## Related literature

For synthetic strategies for the synthesis of carbazole and its derivatives, see: Chakraborty (1993 ▶). For the isolation of pyran­ocarbazoles from various plant species, see: Knölker & Reddy (2002 ▶, and references therein). For the synthesis of related compounds, see: Kavitha & Rajendra Prasad (2003*a* ▶,*b* ▶); Patel (1982 ▶). For the structure of the second product of the reaction yielding the title compound, see: Sridharan *et al.* (2008 ▶).
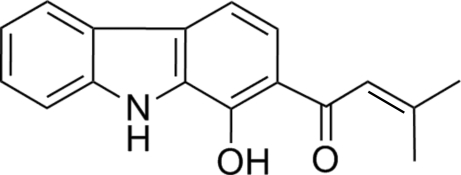


## Experimental

### 

#### Crystal data

C_17_H_15_NO_2_
*M* *_r_* = 265.30
Triclinic, 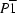

*a* = 6.3416 (9) Å
*b* = 15.202 (2) Å
*c* = 15.462 (3) Å
α = 115.216 (5)°
β = 95.042 (5)°
γ = 101.922 (4)°
*V* = 1293.2 (4) Å^3^
*Z* = 4
Mo *K*α radiation
μ = 0.09 mm^−1^
*T* = 100 K
0.31 × 0.19 × 0.16 mm

#### Data collection

Bruker SMART APEX CCD diffractometer
Absorption correction: multi-scan (*APEX2*; Bruker, 2007 ▶) *T*_min_ = 0.749, *T*_max_ = 0.986
13387 measured reflections
6364 independent reflections
4788 reflections with *I* > 2σ(*I*)
*R*_int_ = 0.026

#### Refinement

*R*[*F* ^2^ > 2σ(*F* ^2^)] = 0.048
*wR*(*F* ^2^) = 0.123
*S* = 1.01
6364 reflections
367 parameters
H-atom parameters constrained
Δρ_max_ = 0.35 e Å^−3^
Δρ_min_ = −0.25 e Å^−3^

### 

Data collection: *APEX2* (Bruker, 2007 ▶); cell refinement: *SAINT* (Bruker, 2007 ▶); data reduction: *SAINT*; program(s) used to solve structure: *SHELXTL* (Sheldrick, 2008 ▶); program(s) used to refine structure: *SHELXTL*; molecular graphics: *SHELXTL* and *Mercury* (Macrae *et al.*, 2008 ▶); software used to prepare material for publication: *SHELXTL*, *PLATON* (Spek, 2009 ▶) and *publCIF* (McMahon & Westrip, 2008 ▶).

## Supplementary Material

Crystal structure: contains datablocks I, global. DOI: 10.1107/S1600536810000322/bv2136sup1.cif

Structure factors: contains datablocks I. DOI: 10.1107/S1600536810000322/bv2136Isup2.hkl

Additional supplementary materials:  crystallographic information; 3D view; checkCIF report

## Figures and Tables

**Table 1:** Hydrogen-bond geometry (Å, °) *Cg*1, *Cg*2 and *Cg*3 are the centroids of the phenyl rings C1*B*–C6*B*, C7*A*–C12*A* and C1*A*–C6*A*, respectively.

*D*—H⋯*A*	*D*—H	H⋯*A*	*D*⋯*A*	*D*—H⋯*A*
O1*B*—H1*D*⋯O2*B*	0.84	1.73	2.4762 (16)	146
O1*A*—H1*C*⋯O2*A*	0.84	1.72	2.4626 (16)	146
N1*B*—H1*B*⋯O1*B*^i^	0.88	2.12	2.9561 (17)	157
N1*A*—H1*A*⋯O1*A*^ii^	0.88	2.08	2.8996 (16)	155
C10*A*—H10*A*⋯*Cg*1^iii^	0.95	2.66	3.365 (2)	132
C10*B*—H10*B*⋯*Cg*2^ii^	0.95	2.68	3.427 (2)	136
C16*A*—H16*A*⋯*Cg*3^iii^	0.95	2.77	3.659 (2)	152
C16*B*—H16*D*⋯*Cg*1^iv^	0.95	2.96	3.846 (2)	151

Symmetry codes: (i) 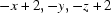; (ii) 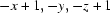; (iii) 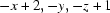; (iv) 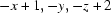.

**Fig. 1.**
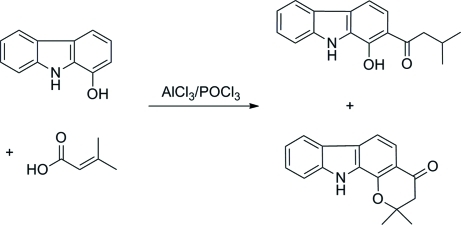
Synthesis of the title compound.

**Fig. 2.**
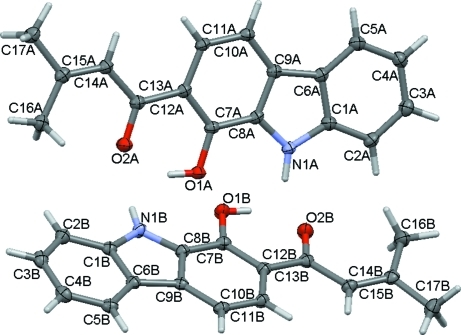
Thermal ellipsoid plot of the two independent molecules with atom numbering scheme. Atomic displacement parameters are at the 50% probablity level.

**Fig. 3.**
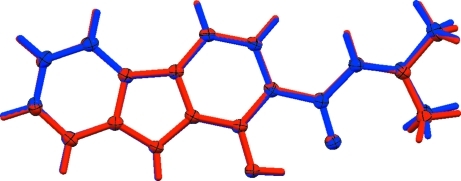
Least square overlay of molecules A (red) and B (blue)

**Fig. 4.**
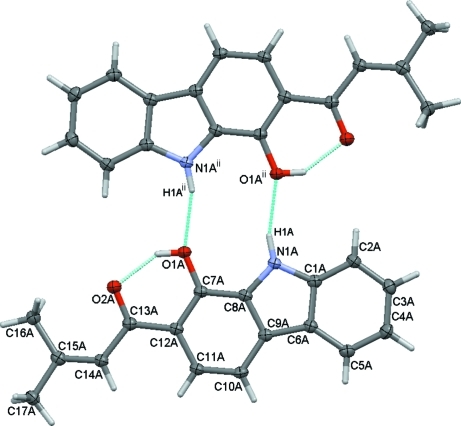
One of the H-bonded dimers. Dashed blue lines respresent hydrogen bonds. Molecule B (not shown) forms dimers with essentially the same geometry. Symmetry operator ii: -x + 1, -y, -z + 1.

**Fig. 5.**
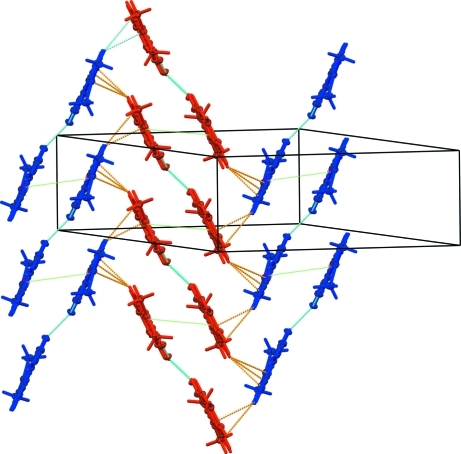
Packing diagram showing the arrangement of molecules and intermolecular interactions. Blue dashed lines: O—H···H and N—H···O hydrogen bonds. Orange dahsed lines: C—H···π interactions. Red dashed lines connect the centroids of π-stacked molecules (see text for details).

## supplementary crystallographic information

### Comment

A number of carbazole alkaloids with intriguing novel structures and useful biological activities were isolated from natural sources over the past decades, which led towards the development of new synthetic strategies for the synthesis of carbazole and its derivatives (Chakraborty, 1993). Among the physiologically active carbazoles found aree pyranocarbazole alkaloids, which have a C-13, C-18 or C-23 framework (Knölker & Reddy, 2002). The basic unit is the C-12 carbazole nucleus with one carbon attached as a methyl, formyl, carboxylic or ester group. This C-13 unit then leads to C-18 or C-23 carbazole alkaloids depending on whether it combines with a hemi-terpenoid or a mono-terpenoid unit. Another observation is that in all the pyranocarbazole derivatives isolated so far, the oxygen atom of the pyran ring is attached to carbon-2 of the carbazole nucleus to form essentially pyrano[3,2-*a*]carbazole as in grinimbine. Patel (1982, and references therein) has reported the synthesis of indolo[3,2-*h*]chromanones from 1-hydroxycarbazoles which were then converted to isomers of grinimbine. Here the yields of compound were reported to be moderate since it was obtained along with the respective 2-acryloyl-1-hydroxycarbazole.

In this context we aimed to prepare pyrano[2,3-*a*]carbazoles using 1-hydroxycarbazoles as starting synthons under various reaction conditions (Kavitha & Rajendra Prasad, 2003*a*,*b*, and references therein). Using the catalyst mixture AlCl_3_/POCl_3_ along with 9-carbazole-1-ol and 3,3-dimethyacrylic acid as the reactants we obatined a mixture of two products *i.e.*, 1-(1-hydroxy-9*H*-carbazol-2-yl)-3-methylbutan-1-one and 2,2-dimethyl-2,3-dihydropyran*o*-[2,3-*a*]carbazol- 4(11*H*)-one as described in an earlier publication (Sridharan *et al.*, 2008) and in Figure 1. The structure of the cyclized compound 2,2-dimethyl-2,3-dihydropyran*o*-[2,3-*a*]carbazol- 4(11*H*)-one was described in the earlier structure report (Sridharan *et al.*, 2008). Here we would like to present the structure of the second compound isolated, 1-(1-hydroxy-9*H*-carbazol-2-yl)-3-methylbutan-1-one.

The title compound crystallizes in a triclinic setting with two crystallographically independent molecules, A and B (Figure 2). The two molecules are virtually superimposable (see overlay of the two structures in Figure 3) but a *PLATON* symmetry check did not reveal any translational or other pseudosymmetry even when using relaxed tolerances (Spek, 2009). Both independent molecules are planar, r.m.s. deviations from planarity are 0.053 and 0.079 Å^2^, respectively, and they are tilted against each other within the structure with a dihedral angle of the planes of the A and B molecules of 53.11 (2)°.

Each molecule exhibits a strong intramolecular O—H···O hydrogen bond between the phenolic hydroxyl group and the keto oxgen atom (Table 1). In addition each type of molecules is connected *via* pairs of N—H···O hydrogen bonds to another molecule of the same type to form centrosymmetric A_2_ and B_2 _dimers (the planes of the dimers are parallel but slightly shifted against each other, Figure 4). The dimers are in turn arranged in offset π-stacks that are extending along the *a* axis direction. The metrics of the interaction are best given for the interaction of the phenol rings C7A to C12A and C7B to C12B with their respective symmetry equivalent counterparts at 2 - *x*, -*y*, 1 - *z* and 1 - *x*, -*y*, 2 - *z*. For these the centroid to centroid distances are 4.083 (1) and 4.089 (1) Å, the interplanar distances are 3.2985 (6) and 3.2992 (7) Å, and the slippages are 2.407 and 2.415 Å, respectively. The offset of the dimers and the tilt angle of the molecules allows for the formation of alternating C—H···π interactions between A and B molecules of parallel stacks. C—H···π interactions are given in Table 1, with ring centroids 1, 2 and 3 being the phenyl rings C1B to C6B, C7A to C12A and C1A to C6A, respectively.

### Experimental

The title compound was synthesized as described previously by Sridharan *et al.* (2008): 9-Carbazole-1-ol (0.001 mol) and 3,3-dimethylacrylic acid (0.001 mol) were dissolved in the mixture of an ice-cold solution of AlCl_3_/POCl_3_ (400 mg/ 6 ml) and kept at room temperature for 24 h. The reaction process as monitored by TLC indicated the formation of two compounds. After completion of the reaction (disappearance of starting material), the residue was poured onto ice water. The solid separated out was filtered, dried and then separated by column chromatography on silica gel using petroleum ether/ ethyl acetate (98:2) as eluents to yield the title compound 1-(1-hydroxy-9*H*-carbazol-2-yl)-3-methylbutan-1-one and 2,2-dimethyl-2,3-dihydropyrano[2,3-*a*]carbazol-4(11*H*)-one, respectively as yellow prisms (Figure 1). The title compound was recrystallized from ethanol. Yield: 0.114 g (43%), m.p. 482- 484 K (209 - 211°C).

### Refinement

Hydrogen atoms were placed in calculated positions with C—H bond distances of 0.95 Å (aromatic H), 0.88 Å (N—H) or 0.84 Å (O—H) and were refined with an isotropic displacement parameter 1.5 (methyl, hydroxyl) or 1.2 times (all others) that of the adjacent carbon or oxygen atom. Methyl and hydroxyl hydrogen atoms were allowed to rotate at fixed angle around the C—C/O bond to best fit the experimental electron density.

### Crystal data

**Table:**

C_17_H_15_NO_2_	*Z* = 4
*M**_r_* = 265.30	*F*(000) = 560
Triclinic, *P*1	*D*_x_ = 1.363 Mg m^−^^3^
Hall symbol: -P 1	Melting point: 483 K
*a* = 6.3416 (9) Å	Mo *K*α radiation, λ = 0.71073 Å
*b* = 15.202 (2) Å	Cell parameters from 3373 reflections
*c* = 15.462 (3) Å	θ = 2.7–29.0°
α = 115.216 (5)°	µ = 0.09 mm^−^^1^
β = 95.042 (5)°	*T* = 100 K
γ = 101.922 (4)°	Plate, orange
*V* = 1293.2 (4) Å^3^	0.31 × 0.19 × 0.16 mm

### Data collection

**Table:**

Bruker SMART APEX CCD diffractometer	6364 independent reflections
Radiation source: fine-focus sealed tube	4788 reflections with *I* > 2σ(*I*)
graphite	*R*_int_ = 0.026
ω scans	θ_max_ = 28.3°, θ_min_ = 1.5°
Absorption correction: multi-scan (*APEX2*; Bruker, 2007)	*h* = −8→8
*T*_min_ = 0.749, *T*_max_ = 0.986	*k* = −20→20
13387 measured reflections	*l* = −20→20

### Refinement

**Table:**

Refinement on *F*^2^	Primary atom site location: structure-invariant direct methods
Least-squares matrix: full	Secondary atom site location: difference Fourier map
*R*[*F*^2^ > 2σ(*F*^2^)] = 0.048	Hydrogen site location: inferred from neighbouring sites
*wR*(*F*^2^) = 0.123	H-atom parameters constrained
*S* = 1.01	*w* = 1/[σ^2^(*F*_o_^2^) + (0.0531*P*)^2^ + 0.5261*P*] where *P* = (*F*_o_^2^ + 2*F*_c_^2^)/3
6364 reflections	(Δ/σ)_max_ < 0.001
367 parameters	Δρ_max_ = 0.35 e Å^−^^3^
0 restraints	Δρ_min_ = −0.25 e Å^−^^3^

### Special details

**Table:**

Geometry. All e.s.d.'s (except the e.s.d. in the dihedral angle between two l.s. planes) are estimated using the full covariance matrix. The cell e.s.d.'s are taken into account individually in the estimation of e.s.d.'s in distances, angles and torsion angles; correlations between e.s.d.'s in cell parameters are only used when they are defined by crystal symmetry. An approximate (isotropic) treatment of cell e.s.d.'s is used for estimating e.s.d.'s involving l.s. planes.
Refinement. Refinement of *F*^2^ against ALL reflections. The weighted *R*-factor *wR* and goodness of fit *S* are based on *F*^2^, conventional *R*-factors *R* are based on *F*, with *F* set to zero for negative *F*^2^. The threshold expression of *F*^2^ > σ(*F*^2^) is used only for calculating *R*-factors(gt) *etc*. and is not relevant to the choice of reflections for refinement. *R*-factors based on *F*^2^ are statistically about twice as large as those based on *F*, and *R*- factors based on ALL data will be even larger.

### Fractional atomic coordinates and isotropic or equivalent isotropic displacement parameters (Å^2^)

**Table:**

	*x*	*y*	*z*	*U*_iso_*/*U*_eq_	
C1A	0.7818 (2)	0.24361 (12)	0.56142 (11)	0.0177 (3)	
C2A	0.6819 (3)	0.31619 (12)	0.61788 (11)	0.0198 (3)	
H2A	0.5434	0.2975	0.6334	0.024*	
C3A	0.7926 (3)	0.41613 (12)	0.65017 (12)	0.0220 (3)	
H3A	0.7278	0.4672	0.6882	0.026*	
C4A	0.9985 (3)	0.44455 (12)	0.62833 (12)	0.0224 (3)	
H4A	1.0709	0.5141	0.6522	0.027*	
C5A	1.0970 (3)	0.37214 (12)	0.57234 (11)	0.0206 (3)	
H5A	1.2366	0.3916	0.5580	0.025*	
C6A	0.9881 (2)	0.26989 (12)	0.53716 (11)	0.0175 (3)	
C7A	0.8360 (2)	−0.00303 (12)	0.40772 (11)	0.0164 (3)	
C8A	0.8533 (2)	0.09890 (12)	0.46513 (11)	0.0165 (3)	
C9A	1.0325 (2)	0.17577 (11)	0.47439 (11)	0.0164 (3)	
C10A	1.2026 (2)	0.15003 (12)	0.42390 (11)	0.0179 (3)	
H10A	1.3252	0.2011	0.4289	0.021*	
C11A	1.1880 (2)	0.04945 (12)	0.36717 (11)	0.0177 (3)	
H11A	1.3029	0.0321	0.3334	0.021*	
C12A	1.0068 (2)	−0.02914 (11)	0.35748 (11)	0.0164 (3)	
C13A	0.9842 (2)	−0.13732 (12)	0.29741 (11)	0.0181 (3)	
C14A	1.1542 (3)	−0.17222 (12)	0.24376 (11)	0.0190 (3)	
H14A	1.2805	−0.1225	0.2493	0.023*	
C15A	1.1436 (3)	−0.26979 (12)	0.18718 (11)	0.0201 (3)	
C16A	0.9526 (3)	−0.35834 (12)	0.16533 (12)	0.0238 (3)	
H16A	0.9365	−0.3639	0.2254	0.036*	
H16B	0.9790	−0.4204	0.1171	0.036*	
H16C	0.8178	−0.3485	0.1392	0.036*	
C17A	1.3362 (3)	−0.29673 (13)	0.14197 (12)	0.0237 (3)	
H17A	1.4516	−0.2348	0.1590	0.036*	
H17B	1.2888	−0.3343	0.0709	0.036*	
H17C	1.3937	−0.3386	0.1665	0.036*	
C1B	0.6941 (2)	−0.19517 (12)	0.77600 (11)	0.0178 (3)	
C2B	0.7881 (3)	−0.27530 (12)	0.73361 (11)	0.0205 (3)	
H2B	0.9299	−0.2729	0.7617	0.025*	
C3B	0.6655 (3)	−0.35811 (12)	0.64911 (12)	0.0224 (3)	
H3B	0.7260	−0.4132	0.6179	0.027*	
C4B	0.4536 (3)	−0.36301 (12)	0.60808 (12)	0.0218 (3)	
H4B	0.3725	−0.4217	0.5508	0.026*	
C5B	0.3619 (3)	−0.28337 (12)	0.65017 (11)	0.0197 (3)	
H5B	0.2188	−0.2870	0.6223	0.024*	
C6B	0.4834 (2)	−0.19745 (12)	0.73445 (11)	0.0171 (3)	
C7B	0.6547 (2)	0.05079 (11)	0.94208 (11)	0.0168 (3)	
C8B	0.6330 (2)	−0.04659 (11)	0.86929 (11)	0.0169 (3)	
C9B	0.4459 (2)	−0.10097 (11)	0.79421 (11)	0.0163 (3)	
C10B	0.2753 (2)	−0.05548 (12)	0.79034 (11)	0.0180 (3)	
H10B	0.1488	−0.0906	0.7394	0.022*	
C11B	0.2948 (2)	0.04032 (12)	0.86139 (11)	0.0181 (3)	
H11B	0.1798	0.0710	0.8586	0.022*	
C12B	0.4818 (2)	0.09561 (11)	0.93938 (11)	0.0168 (3)	
C13B	0.5056 (2)	0.19747 (12)	1.01756 (11)	0.0182 (3)	
C14B	0.3307 (2)	0.24795 (12)	1.02043 (11)	0.0192 (3)	
H14B	0.1949	0.2088	0.9761	0.023*	
C15B	0.3472 (3)	0.34515 (12)	1.08080 (12)	0.0205 (3)	
C16B	0.5470 (3)	0.41995 (13)	1.15485 (13)	0.0277 (4)	
H16D	0.5608	0.4072	1.2118	0.042*	
H16E	0.5326	0.4885	1.1750	0.042*	
H16F	0.6781	0.4130	1.1262	0.042*	
C17B	0.1508 (3)	0.38542 (13)	1.07778 (13)	0.0267 (4)	
H17D	0.0296	0.3324	1.0266	0.040*	
H17E	0.1892	0.4432	1.0637	0.040*	
H17F	0.1055	0.4070	1.1410	0.040*	
N1A	0.7042 (2)	0.13977 (10)	0.51833 (9)	0.0180 (3)	
H1A	0.5806	0.1055	0.5239	0.022*	
N1B	0.7808 (2)	−0.10416 (10)	0.85829 (9)	0.0182 (3)	
H1B	0.9085	−0.0858	0.8972	0.022*	
O1A	0.65515 (17)	−0.07178 (8)	0.40157 (8)	0.0203 (2)	
H1C	0.6662	−0.1302	0.3672	0.030*	
O2A	0.81641 (18)	−0.20127 (8)	0.29274 (8)	0.0235 (3)	
O1B	0.83971 (17)	0.09766 (8)	1.01146 (8)	0.0207 (2)	
H1D	0.8316	0.1547	1.0516	0.031*	
O2B	0.67656 (18)	0.24120 (8)	1.08281 (8)	0.0233 (3)	

### Atomic displacement parameters (Å^2^)

**Table:**

	*U*^11^	*U*^22^	*U*^33^	*U*^12^	*U*^13^	*U*^23^
C1A	0.0170 (7)	0.0206 (8)	0.0164 (7)	0.0041 (6)	0.0024 (6)	0.0099 (6)
C2A	0.0189 (7)	0.0237 (8)	0.0185 (8)	0.0080 (6)	0.0058 (6)	0.0097 (7)
C3A	0.0249 (8)	0.0231 (8)	0.0192 (8)	0.0095 (7)	0.0050 (6)	0.0093 (7)
C4A	0.0245 (8)	0.0185 (8)	0.0237 (8)	0.0043 (6)	0.0023 (6)	0.0103 (7)
C5A	0.0184 (7)	0.0236 (8)	0.0207 (8)	0.0043 (6)	0.0033 (6)	0.0115 (7)
C6A	0.0170 (7)	0.0210 (8)	0.0166 (7)	0.0059 (6)	0.0031 (6)	0.0103 (6)
C7A	0.0139 (7)	0.0206 (8)	0.0159 (7)	0.0037 (6)	0.0027 (6)	0.0100 (6)
C8A	0.0149 (7)	0.0214 (8)	0.0153 (7)	0.0059 (6)	0.0034 (6)	0.0097 (6)
C9A	0.0155 (7)	0.0205 (8)	0.0149 (7)	0.0039 (6)	0.0015 (6)	0.0102 (6)
C10A	0.0153 (7)	0.0210 (8)	0.0192 (8)	0.0032 (6)	0.0036 (6)	0.0117 (6)
C11A	0.0149 (7)	0.0228 (8)	0.0183 (7)	0.0060 (6)	0.0056 (6)	0.0112 (6)
C12A	0.0156 (7)	0.0204 (8)	0.0152 (7)	0.0056 (6)	0.0028 (6)	0.0097 (6)
C13A	0.0168 (7)	0.0213 (8)	0.0172 (7)	0.0045 (6)	0.0025 (6)	0.0101 (6)
C14A	0.0176 (7)	0.0213 (8)	0.0197 (8)	0.0058 (6)	0.0052 (6)	0.0101 (7)
C15A	0.0201 (7)	0.0252 (8)	0.0181 (8)	0.0082 (6)	0.0040 (6)	0.0117 (7)
C16A	0.0226 (8)	0.0212 (8)	0.0264 (9)	0.0071 (6)	0.0061 (7)	0.0088 (7)
C17A	0.0218 (8)	0.0263 (9)	0.0233 (8)	0.0096 (7)	0.0065 (6)	0.0098 (7)
C1B	0.0183 (7)	0.0198 (8)	0.0167 (7)	0.0043 (6)	0.0047 (6)	0.0097 (6)
C2B	0.0215 (8)	0.0223 (8)	0.0208 (8)	0.0077 (6)	0.0059 (6)	0.0114 (7)
C3B	0.0302 (9)	0.0203 (8)	0.0210 (8)	0.0097 (7)	0.0098 (7)	0.0111 (7)
C4B	0.0268 (8)	0.0187 (8)	0.0167 (8)	0.0022 (6)	0.0041 (6)	0.0071 (6)
C5B	0.0191 (7)	0.0232 (8)	0.0174 (7)	0.0033 (6)	0.0038 (6)	0.0107 (7)
C6B	0.0168 (7)	0.0194 (8)	0.0175 (7)	0.0050 (6)	0.0057 (6)	0.0103 (6)
C7B	0.0151 (7)	0.0200 (8)	0.0151 (7)	0.0031 (6)	0.0016 (6)	0.0090 (6)
C8B	0.0154 (7)	0.0201 (8)	0.0177 (7)	0.0054 (6)	0.0041 (6)	0.0104 (6)
C9B	0.0164 (7)	0.0184 (7)	0.0145 (7)	0.0028 (6)	0.0043 (6)	0.0083 (6)
C10B	0.0148 (7)	0.0226 (8)	0.0173 (7)	0.0040 (6)	0.0014 (6)	0.0106 (6)
C11B	0.0157 (7)	0.0216 (8)	0.0194 (8)	0.0068 (6)	0.0028 (6)	0.0109 (6)
C12B	0.0171 (7)	0.0187 (8)	0.0168 (7)	0.0054 (6)	0.0047 (6)	0.0098 (6)
C13B	0.0186 (7)	0.0196 (8)	0.0179 (7)	0.0049 (6)	0.0050 (6)	0.0097 (6)
C14B	0.0169 (7)	0.0223 (8)	0.0184 (8)	0.0055 (6)	0.0026 (6)	0.0093 (7)
C15B	0.0205 (8)	0.0233 (8)	0.0208 (8)	0.0071 (6)	0.0080 (6)	0.0116 (7)
C16B	0.0222 (8)	0.0213 (9)	0.0325 (10)	0.0055 (7)	0.0056 (7)	0.0060 (8)
C17B	0.0264 (9)	0.0265 (9)	0.0246 (9)	0.0128 (7)	0.0041 (7)	0.0071 (7)
N1A	0.0156 (6)	0.0186 (6)	0.0200 (7)	0.0046 (5)	0.0070 (5)	0.0085 (5)
N1B	0.0145 (6)	0.0198 (7)	0.0187 (6)	0.0060 (5)	0.0013 (5)	0.0071 (5)
O1A	0.0173 (5)	0.0181 (5)	0.0243 (6)	0.0028 (4)	0.0085 (4)	0.0087 (5)
O2A	0.0206 (6)	0.0208 (6)	0.0263 (6)	0.0032 (5)	0.0082 (5)	0.0085 (5)
O1B	0.0179 (5)	0.0194 (6)	0.0194 (6)	0.0052 (4)	−0.0021 (4)	0.0048 (5)
O2B	0.0209 (6)	0.0205 (6)	0.0226 (6)	0.0048 (5)	−0.0020 (5)	0.0060 (5)

### Geometric parameters (Å, °)

**Table:**

C1A—N1A	1.380 (2)	C1B—C6B	1.418 (2)
C1A—C2A	1.395 (2)	C2B—C3B	1.383 (2)
C1A—C6A	1.418 (2)	C2B—H2B	0.9500
C2A—C3A	1.379 (2)	C3B—C4B	1.408 (2)
C2A—H2A	0.9500	C3B—H3B	0.9500
C3A—C4A	1.406 (2)	C4B—C5B	1.383 (2)
C3A—H3A	0.9500	C4B—H4B	0.9500
C4A—C5A	1.385 (2)	C5B—C6B	1.398 (2)
C4A—H4A	0.9500	C5B—H5B	0.9500
C5A—C6A	1.400 (2)	C6B—C9B	1.446 (2)
C5A—H5A	0.9500	C7B—O1B	1.3478 (17)
C6A—C9A	1.449 (2)	C7B—C8B	1.395 (2)
C7A—O1A	1.3479 (17)	C7B—C12B	1.412 (2)
C7A—C8A	1.393 (2)	C8B—N1B	1.3833 (19)
C7A—C12A	1.414 (2)	C8B—C9B	1.405 (2)
C8A—N1A	1.3786 (19)	C9B—C10B	1.409 (2)
C8A—C9A	1.399 (2)	C10B—C11B	1.372 (2)
C9A—C10A	1.410 (2)	C10B—H10B	0.9500
C10A—C11A	1.378 (2)	C11B—C12B	1.428 (2)
C10A—H10A	0.9500	C11B—H11B	0.9500
C11A—C12A	1.421 (2)	C12B—C13B	1.469 (2)
C11A—H11A	0.9500	C13B—O2B	1.2545 (19)
C12A—C13A	1.472 (2)	C13B—C14B	1.467 (2)
C13A—O2A	1.2577 (18)	C14B—C15B	1.345 (2)
C13A—C14A	1.466 (2)	C14B—H14B	0.9500
C14A—C15A	1.347 (2)	C15B—C16B	1.500 (2)
C14A—H14A	0.9500	C15B—C17B	1.502 (2)
C15A—C17A	1.503 (2)	C16B—H16D	0.9800
C15A—C16A	1.504 (2)	C16B—H16E	0.9800
C16A—H16A	0.9800	C16B—H16F	0.9800
C16A—H16B	0.9800	C17B—H17D	0.9800
C16A—H16C	0.9800	C17B—H17E	0.9800
C17A—H17A	0.9800	C17B—H17F	0.9800
C17A—H17B	0.9800	N1A—H1A	0.8800
C17A—H17C	0.9800	N1B—H1B	0.8800
C1B—N1B	1.378 (2)	O1A—H1C	0.8400
C1B—C2B	1.399 (2)	O1B—H1D	0.8400
			
N1A—C1A—C2A	128.77 (14)	C3B—C2B—H2B	121.5
N1A—C1A—C6A	108.97 (13)	C1B—C2B—H2B	121.5
C2A—C1A—C6A	122.23 (14)	C2B—C3B—C4B	121.82 (15)
C3A—C2A—C1A	117.24 (15)	C2B—C3B—H3B	119.1
C3A—C2A—H2A	121.4	C4B—C3B—H3B	119.1
C1A—C2A—H2A	121.4	C5B—C4B—C3B	120.82 (15)
C2A—C3A—C4A	121.82 (15)	C5B—C4B—H4B	119.6
C2A—C3A—H3A	119.1	C3B—C4B—H4B	119.6
C4A—C3A—H3A	119.1	C4B—C5B—C6B	118.86 (15)
C5A—C4A—C3A	120.66 (15)	C4B—C5B—H5B	120.6
C5A—C4A—H4A	119.7	C6B—C5B—H5B	120.6
C3A—C4A—H4A	119.7	C5B—C6B—C1B	119.39 (14)
C4A—C5A—C6A	119.11 (15)	C5B—C6B—C9B	133.95 (14)
C4A—C5A—H5A	120.4	C1B—C6B—C9B	106.65 (13)
C6A—C5A—H5A	120.4	O1B—C7B—C8B	118.52 (13)
C5A—C6A—C1A	118.91 (14)	O1B—C7B—C12B	123.14 (14)
C5A—C6A—C9A	134.61 (14)	C8B—C7B—C12B	118.34 (13)
C1A—C6A—C9A	106.44 (13)	N1B—C8B—C7B	127.82 (14)
O1A—C7A—C8A	118.30 (13)	N1B—C8B—C9B	109.87 (13)
O1A—C7A—C12A	123.26 (14)	C7B—C8B—C9B	122.31 (14)
C8A—C7A—C12A	118.43 (13)	C8B—C9B—C10B	119.36 (14)
N1A—C8A—C7A	127.35 (14)	C8B—C9B—C6B	106.02 (13)
N1A—C8A—C9A	110.20 (13)	C10B—C9B—C6B	134.60 (14)
C7A—C8A—C9A	122.44 (14)	C11B—C10B—C9B	118.89 (14)
C8A—C9A—C10A	119.29 (14)	C11B—C10B—H10B	120.6
C8A—C9A—C6A	106.02 (13)	C9B—C10B—H10B	120.6
C10A—C9A—C6A	134.66 (14)	C10B—C11B—C12B	122.35 (14)
C11A—C10A—C9A	118.85 (14)	C10B—C11B—H11B	118.8
C11A—C10A—H10A	120.6	C12B—C11B—H11B	118.8
C9A—C10A—H10A	120.6	C7B—C12B—C11B	118.72 (14)
C10A—C11A—C12A	122.25 (14)	C7B—C12B—C13B	117.64 (13)
C10A—C11A—H11A	118.9	C11B—C12B—C13B	123.64 (14)
C12A—C11A—H11A	118.9	O2B—C13B—C14B	119.83 (14)
C7A—C12A—C11A	118.74 (14)	O2B—C13B—C12B	119.54 (14)
C7A—C12A—C13A	117.25 (13)	C14B—C13B—C12B	120.63 (14)
C11A—C12A—C13A	124.01 (14)	C15B—C14B—C13B	125.25 (15)
O2A—C13A—C14A	119.28 (14)	C15B—C14B—H14B	117.4
O2A—C13A—C12A	119.28 (14)	C13B—C14B—H14B	117.4
C14A—C13A—C12A	121.43 (13)	C14B—C15B—C16B	125.88 (15)
C15A—C14A—C13A	124.55 (14)	C14B—C15B—C17B	119.22 (15)
C15A—C14A—H14A	117.7	C16B—C15B—C17B	114.89 (14)
C13A—C14A—H14A	117.7	C15B—C16B—H16D	109.5
C14A—C15A—C17A	119.65 (15)	C15B—C16B—H16E	109.5
C14A—C15A—C16A	125.41 (15)	H16D—C16B—H16E	109.5
C17A—C15A—C16A	114.93 (14)	C15B—C16B—H16F	109.5
C15A—C16A—H16A	109.5	H16D—C16B—H16F	109.5
C15A—C16A—H16B	109.5	H16E—C16B—H16F	109.5
H16A—C16A—H16B	109.5	C15B—C17B—H17D	109.5
C15A—C16A—H16C	109.5	C15B—C17B—H17E	109.5
H16A—C16A—H16C	109.5	H17D—C17B—H17E	109.5
H16B—C16A—H16C	109.5	C15B—C17B—H17F	109.5
C15A—C17A—H17A	109.5	H17D—C17B—H17F	109.5
C15A—C17A—H17B	109.5	H17E—C17B—H17F	109.5
H17A—C17A—H17B	109.5	C8A—N1A—C1A	108.35 (12)
C15A—C17A—H17C	109.5	C8A—N1A—H1A	125.8
H17A—C17A—H17C	109.5	C1A—N1A—H1A	125.8
H17B—C17A—H17C	109.5	C1B—N1B—C8B	108.43 (12)
N1B—C1B—C2B	128.95 (14)	C1B—N1B—H1B	125.8
N1B—C1B—C6B	109.01 (13)	C8B—N1B—H1B	125.8
C2B—C1B—C6B	122.02 (14)	C7A—O1A—H1C	109.5
C3B—C2B—C1B	117.05 (15)	C7B—O1B—H1D	109.5
			
N1A—C1A—C2A—C3A	−178.05 (15)	C3B—C4B—C5B—C6B	0.0 (2)
C6A—C1A—C2A—C3A	−0.2 (2)	C4B—C5B—C6B—C1B	−1.7 (2)
C1A—C2A—C3A—C4A	−0.6 (2)	C4B—C5B—C6B—C9B	176.91 (15)
C2A—C3A—C4A—C5A	0.5 (2)	N1B—C1B—C6B—C5B	−179.42 (13)
C3A—C4A—C5A—C6A	0.3 (2)	C2B—C1B—C6B—C5B	2.0 (2)
C4A—C5A—C6A—C1A	−1.1 (2)	N1B—C1B—C6B—C9B	1.62 (16)
C4A—C5A—C6A—C9A	176.35 (16)	C2B—C1B—C6B—C9B	−176.96 (14)
N1A—C1A—C6A—C5A	179.28 (13)	O1B—C7B—C8B—N1B	0.3 (2)
C2A—C1A—C6A—C5A	1.1 (2)	C12B—C7B—C8B—N1B	−179.84 (14)
N1A—C1A—C6A—C9A	1.17 (16)	O1B—C7B—C8B—C9B	180.00 (13)
C2A—C1A—C6A—C9A	−177.06 (14)	C12B—C7B—C8B—C9B	−0.1 (2)
O1A—C7A—C8A—N1A	0.1 (2)	N1B—C8B—C9B—C10B	−178.81 (13)
C12A—C7A—C8A—N1A	178.99 (14)	C7B—C8B—C9B—C10B	1.4 (2)
O1A—C7A—C8A—C9A	−178.67 (13)	N1B—C8B—C9B—C6B	0.17 (17)
C12A—C7A—C8A—C9A	0.2 (2)	C7B—C8B—C9B—C6B	−179.61 (13)
N1A—C8A—C9A—C10A	−178.80 (13)	C5B—C6B—C9B—C8B	−179.81 (16)
C7A—C8A—C9A—C10A	0.2 (2)	C1B—C6B—C9B—C8B	−1.08 (16)
N1A—C8A—C9A—C6A	−0.38 (16)	C5B—C6B—C9B—C10B	−1.1 (3)
C7A—C8A—C9A—C6A	178.57 (13)	C1B—C6B—C9B—C10B	177.67 (16)
C5A—C6A—C9A—C8A	−178.16 (16)	C8B—C9B—C10B—C11B	−1.3 (2)
C1A—C6A—C9A—C8A	−0.48 (16)	C6B—C9B—C10B—C11B	−179.88 (15)
C5A—C6A—C9A—C10A	−0.1 (3)	C9B—C10B—C11B—C12B	−0.2 (2)
C1A—C6A—C9A—C10A	177.58 (16)	O1B—C7B—C12B—C11B	178.60 (14)
C8A—C9A—C10A—C11A	−0.4 (2)	C8B—C7B—C12B—C11B	−1.3 (2)
C6A—C9A—C10A—C11A	−178.27 (15)	O1B—C7B—C12B—C13B	−1.2 (2)
C9A—C10A—C11A—C12A	0.3 (2)	C8B—C7B—C12B—C13B	178.92 (13)
O1A—C7A—C12A—C11A	178.50 (13)	C10B—C11B—C12B—C7B	1.5 (2)
C8A—C7A—C12A—C11A	−0.3 (2)	C10B—C11B—C12B—C13B	−178.77 (14)
O1A—C7A—C12A—C13A	−1.2 (2)	C7B—C12B—C13B—O2B	0.6 (2)
C8A—C7A—C12A—C13A	180.00 (13)	C11B—C12B—C13B—O2B	−179.21 (14)
C10A—C11A—C12A—C7A	0.1 (2)	C7B—C12B—C13B—C14B	−178.87 (14)
C10A—C11A—C12A—C13A	179.72 (14)	C11B—C12B—C13B—C14B	1.4 (2)
C7A—C12A—C13A—O2A	0.3 (2)	O2B—C13B—C14B—C15B	9.6 (2)
C11A—C12A—C13A—O2A	−179.31 (14)	C12B—C13B—C14B—C15B	−170.95 (15)
C7A—C12A—C13A—C14A	−178.98 (13)	C13B—C14B—C15B—C16B	1.0 (3)
C11A—C12A—C13A—C14A	1.4 (2)	C13B—C14B—C15B—C17B	−177.81 (15)
O2A—C13A—C14A—C15A	1.0 (2)	C7A—C8A—N1A—C1A	−177.76 (14)
C12A—C13A—C14A—C15A	−179.66 (15)	C9A—C8A—N1A—C1A	1.12 (17)
C13A—C14A—C15A—C17A	−175.27 (14)	C2A—C1A—N1A—C8A	176.66 (15)
C13A—C14A—C15A—C16A	3.4 (3)	C6A—C1A—N1A—C8A	−1.42 (16)
N1B—C1B—C2B—C3B	−178.80 (15)	C2B—C1B—N1B—C8B	176.92 (15)
C6B—C1B—C2B—C3B	−0.5 (2)	C6B—C1B—N1B—C8B	−1.54 (17)
C1B—C2B—C3B—C4B	−1.2 (2)	C7B—C8B—N1B—C1B	−179.39 (14)
C2B—C3B—C4B—C5B	1.5 (2)	C9B—C8B—N1B—C1B	0.85 (17)

### Hydrogen-bond geometry (Å, °)

**Table:**

Cg1, Cg2 and Cg3 are the centroids of the phenyl rings C1B–C6B, C7A–C12A and C1A–C6A, respectively.

**Table:**

*D*—H···*A*	*D*—H	H···*A*	*D*···*A*	*D*—H···*A*
O1B—H1D···O2B	0.84	1.73	2.4762 (16)	146
O1A—H1C···O2A	0.84	1.72	2.4626 (16)	146
N1B—H1B···O1B^i^	0.88	2.12	2.9561 (17)	157
N1A—H1A···O1A^ii^	0.88	2.08	2.8996 (16)	155
C10A—H10A···Cg1^iii^	0.95	2.66	3.365 (2)	132
C10B—H10B···Cg2^ii^	0.95	2.68	3.427 (2)	136
C16A—H16A···Cg3^iii^	0.95	2.77	3.659 (2)	152
C16B—H16D···Cg1^iv^	0.95	2.96	3.846 (2)	151

Symmetry codes: (i) −*x*+2, −*y*, −*z*+2; (ii) −*x*+1, −*y*, −*z*+1; (iii) −*x*+2, −*y*, −*z*+1; (iv) −*x*+1, −*y*, −*z*+2.
